# Clonal spread of antimicrobial-resistant *Escherichia coli *isolates among pups in two kennels

**DOI:** 10.1186/1751-0147-53-11

**Published:** 2011-02-17

**Authors:** Kazuki Harada, Erika Morimoto, Yasushi Kataoka, Toshio Takahashi

**Affiliations:** 1 Laboratory of Veterinary Microbiology, Nippon Veterinary and Life Science University, 1-7-1, Kyonan-cho, Musashino, Tokyo 180-8602, Japan

## Abstract

Although the dog breeding industry is common in many countries, the presence of antimicrobial resistant bacteria among pups in kennels has been infrequently investigated. This study was conducted to better understand the epidemiology of antimicrobial-resistant *Escherichia coli *isolates from kennel pups not treated with antimicrobials. We investigated susceptibilities to 11 antimicrobials, and prevalence of extended-spectrum β-lactamase (ESBL) in 86 faecal *E. coli *isolates from 43 pups in two kennels. Genetic relatedness among all isolates was assessed using pulsed-field gel electrophoresis (PFGE). Susceptibility tests revealed that 76% of the isolates were resistant to one or more of tested antimicrobials, with resistance to dihydrostreptomycin most frequently encountered (66.3%) followed by ampicillin (60.5%), trimethoprim-sulfamethoxazole (41.9%), oxytetracycline (26.7%), and chloramphenicol (26.7%). Multidrug resistance, defined as resistance against two or more classes of antimicrobials, was observed in 52 (60.5%) isolates. Three pups in one kennel harboured SHV-12 ESBL-producing isolates. A comparison between the two kennels showed that frequencies of resistance against seven antimicrobials and the variation in resistant phenotypes differed significantly. Analysis by PFGE revealed that clone sharing rates among pups of the same litters were not significantly different in both kennels (64.0% *vs. *88.9%), whereas the rates among pups from different litters were significantly different between the two kennels (72.0% *vs. *33.3%, *P *< 0.05). The pups in the two kennels had antimicrobial-resistant *E. coli *clones, including multidrug-resistant and ESBL-producing clones. It is likely that resistant and susceptible bacteria can clonally spread among the same and/or different litters thus affecting the resistance prevalence.

## Findings

Spread of antimicrobial-resistant bacteria from companion animals to humans causes concern but the role of companion animals as reservoirs of antimicrobial resistant bacteria requires further investigation [1]. *Escherichia coli *are commonly found in the intestinal tract of animals, including dogs, and constitute a reservoir of resistant genes for potentially pathogenic bacteria [1]. Dogs may also be a reservoir of *E. coli *strains that cause extraintestinal infections in humans [2]. Antimicrobial resistance of canine *E. coli *has previously been investigated [3-5].

Antimicrobial-resistant *E. coli *have been isolated more frequently in kennel dogs than in individually owned dogs [3]. Breeding of multiple dogs at one location may increase the risk of spreading antimicrobial resistant clones in the population similar to livestock on the same premises [6,7].

The purpose of the present study was to compare phenotypic and genetic characteristics of antimicrobial resistant *E. coli *isolated from the faeces of pups in kennels, and to investigate genetic relatedness among these isolates as the epidemiology of antimicrobial resistant bacteria in dog populations has not been extensively studied.

Faecal samples were obtained from 43 apparently healthy pups not above two months of age from two kennels (A and B). Twenty-five pups were from eight litters in kennel A (A-a to A-h), and 18 were from five litters in kennel B (B-a to B-e; Table 1). There was no history of antimicrobial use in the pups but the dams may have been administered lincomycin for postpartum infection prophylaxis. Briefly, faecal swabs were plated onto desoxycholate-hydrogen sulphide-lactose agar (Eiken Chemical, Tochigi, Japan) and incubated overnight at 37°C in an aerobic atmosphere. Subsequently, two lactose fermenting colonies with typical *E. coli *morphology were picked and subjected to confirmatory Gram staining, indole, methyl red, Voges-Proskauer, and Simmons' Citrate tests.

**Table 1:** Details of surveyed pups in this study.

Kennel	Litter	Breed	No. of pups	Pup identity	Date of birth	Age in days
A	A-a	Toy poodle	3	A-a-1 - A-a-3	5/13/2009	30
	A-b	Toy poodle	3	A-b-1 - A-b-3	5/15/2009	28
	A-c	Chihuahua	2	A-c-1 - A-c-2	4/17/2009	57
	A-d	Toy poodle	3	A-d-1 - A-d-3	6/23/2009	45
	A-e	Toy poodle	4	A-e-1 - A-e-4	7/1/2009	37
	A-f	Toy poodle	5	A-f-1 - A-f-5	7/9/2009	29
	A-g	Toy poodle	3	A-g-1-A-g-3	9/2/2009	38
	A-h	Toy poodle	2	A-h-1 - A-h-2	9/6/2009	35
	Total		25			

B	B-a	Maltese	2	B-a-1 - B-a-2	8/3/2009	46
	B-b	Beagle	4	B-b-1 - B-b-4	7/29/2009	51
	B-c	Miniature dachshund	5	B-c-1 - B-c-5	8/1/2009	51
	B-d	Papillon	3	B-d-1 - B-d-3	8/17/2009	53
	B-e	Chihuahua	4	B-e-1 - B-e-4	8/25/2009	55
	Total		18			

Informed consent from both kennels was obtained and treated in accordance with the Japanese Law Concerning the Protection of Personal Information (Law No. 57, 2003).

Minimum inhibitory concentrations of ampicillin (AMP), cefazolin, ceftiofur, dihydrostreptomycin (DHS), gentamicin, kanamycin, oxytetracycline (OTC), chloramphenicol (CHL), trimethoprim-sulfamethoxazole (SXT), nalidixic acid and enrofloxacin were determined using the agar dilution method according to the Clinical and Laboratory Standards Institute (CLSI) Guidelines [8]. CLSI resistance breakpoints [8,9] were used in the categorical analysis of all drugs except DHS where 32 μg/mL was used as reported elsewhere [10]. For quality control, *E. coli *ATCC 25922 was used.

All isolates were screened for extended-spectrum β-lactamase (ESBL) production by the combination disk test (cefotaxime and ceftazidime with or without clavulanic acid) [8]. ESBL-producing strains were examined for β-lactamase-encoding genes including *bla*TEM, *bla*SHV, *bla*CTX-M-2 group, and *bla*CTX-M-9 group by polymerase chain reaction and sequencing [11].

Pulsed-field gel electrophoresis (PFGE) was performed according to standard methods outlined by PulseNet [12]. DNA was digested with *Xba*I (Takara, Shiga, Japan) and electrophoresed on a CHEF DRII (Bio-Rad Laboratories, Hercules, CA, USA), with switch times of 2.2-54.2 s at 14°C for 20 h. PFGE profiles were analysed using BioNumerics software version 4.0 (Applied Maths, TX, USA). DNA fragments on each gel were normalised using the λ molecular weight marker on each gel to enable comparisons between different gels. Cluster analysis was performed by the unweighted pair group method using arithmetic average. DNA relatedness was calculated based on the Dice coefficient.

The prevalence of antimicrobial resistance and rates of clone sharing between the two kennels were compared using Fisher's exact test.

In this study, we investigated the prevalence of antimicrobial resistance in faecal *E. coli *isolates from kennel pups without a history of antimicrobial use; however, their dams may have been administered with lincomycin. If this is the case, the possibility that the pups were exposed to the agent via the milk cannot be excluded. It is generally known that lincomycin is inactive against aerobic Gram-negative bacteria including *E. coli*, but active against Gram-positive and/or anaerobic bacteria [13], possibly causing changes in the intestinal microflora composition. The effects of an altered intestinal flora on the population of resistant and susceptible *E. coli *are unknown, but may need to be taken into account in the present results.

We found that 75.6% (*n *= 65) of *E. coli *isolates originating from 35 pups in two kennels, were resistant to one or more of the antimicrobials tested. Resistance to DHS was most frequent (66.3%), followed by AMP (60.5%), SXT (41.9%), OTC (26.7%), and CHL (26.7%) (Table 2). Multidrug resistance defined as resistance against two or more classes of antimicrobials was observed in 60.5% (*n *= 52) of isolates, originating from 29 pups in two kennels. Comparison between kennels A and B revealed that the prevalence of resistance against seven of the tested antimicrobials differed significantly (*P *< 0.05). Twelve and five resistance patterns were observed in kennels A and B, respectively, with the patterns differing between the two kennels, except for the AMP-DHS-SXT and DHS resistance phenotypes (Table 3). These findings indicate that the prevalence of antimicrobial resistant *E. coli *in pups varies between kennels.

**Table 2:** The minimum inhibitory concentration (MIC) range and resistance rates among *Escherichia coli *isolates from pups originating from two kennels (A and B).

**Substance**^**a**^	MIC range (μg/mL)	**MIC**_**50**_	**MIC**_**90**_	**Resistance breakpoints (μg/mL)**^**b**^	No. of resistant isolates (%)/No. of pups that harboured resistant isolate(s) (%)		
					
					Total	Kennel A	Kennel B
					(n = 86/43)	(n = 50/25)	(n = 36/18)
AMP	2 - >512	>512	>512	≥32	52 (60.5)/30 (69.8)	29 (58.0)/17 (68.0)	23 (63.9)/13 (72.2)
CFZ	8 - 128	4	8	≥32	5 (5.8)/3 (7.0)	0 (0)/0 (0)	5 (13.9)*/3 (16.7)
CEF	≤0.125 - 32	0.5	1	≥8	5 (5.8)/3 (7.0)	0 (0)/0 (0)	5 (13.9)*/3 (16.7)
DHS	2 - >512	512	>512	≥32	57 (66.3)/32 (74.4)	35 (70.0)/19 (76.0)	22 (61.1)/13 (72.2)
GEN	0.5 - 256	1	128	≥16	16 (18.6)/12 (27.9)	16 (32.0)*/12 (48.0)*	0 (0)/0 (0)
KAN	2 - >512	4	16	≥64	3 (3.5)/2 (4.7)	3 (6.0)/2 (8.0)	0 (0)/0 (0)
OTC	1 - 512	2	512	≥16	23 (26.7)/17 (39.5)	18 (36.0)*/14 (56.0)*	5 (13.9)/3 (16.7)
CHL	4 - >512	8	512	≥32	23 (26.7)/17 (39.5)	18 (36.0)*/14 (56.0)*	5 (13.9)/3 (16.7)
NAL	2 - >512	4	16	≥32	7 (8.1)/5 (11.6)	2 (4.0)/2 (8.0)	5 (13.9)/3 (16.7)
ENR	≤0.03 - 256	0.06	1	≥4	5 (5.8)/3 (7.0)	0 (0)/0 (0)	5 (13.9)*/3 (16.7)
SXT	≤0.25/4.75 - >64/1216	1/19	>64/1216	≥16/304	36 (41.9)/21 (48.8)	27 (54.0)*/16 (64.0)*	9 (25.0)/5 (27.8)

^a^AMP, ampicillin; CFZ, cefazolin; CEF, ceftiofur; DHS, dihydrostreptomycin; GEN, gentamicin; KAN, kanamycin; OTC, oxytetracycline; CHL, chloramphenicol; NAL, nalidixic acid; ENR, enrofloxacin; SXT, trimethoprim-sulfamethoxazole.

^b^The breakpoints for AMP, CFZ, CEF, GEN, KAN, OTC, CHL, ENR and SXT, and that for NAL were based on CLSI document M31-A3 [9] and M100-S20 [8], respectively, whereas the breakpoint of DHS was based on an epidemiological cut-off value according to another report [10]

**Table 3:** The distribution of resistance phenotypes among *Escherichiacoli *isolates from pups in two kennels (A and B).

**Resistance patterns**^**a**^	No. of resistant isolates (%)/No. of pups that harboured resistant isolate (s) (%)		
	Total	Kennel A	Kennel B
	
	(n = 86/43)	(n = 50/25)	(n = 36/18)
AMP-CFZ-CFT-OTC-CHL-NAL-ENR	5 (5.8)/3 (7.0)	0 (0)/0 (0)	5 (13.9)/3 (16.7)
AMP-DHS-GEN-KAN-OTC-CHL-SXT	3 (3.5)/2 (4.7)	3 (6.0)/2 (8.0)	0 (0)/0 (0)
AMP-DHS-GEN-OTC-CHL-NAL-SXT	1 (1.2)/1 (2.3)	1 (2.0)/1 (4.0)	0 (0)/0 (0)
AMP-DHS-GEN-OTC-CHL-SXT	11 (12.8)/9 (20.9)	11 (22.0)/9 (36.0)	0 (0)/0 (0)
AMP-DHS-GEN-CHL-SXT	1 (1.2)/1 (2.3)	1 (2.0)/1 (4.0)	0 (0)/0 (0)
AMP-DHS-OTC-SXT	2 (2.3)/1 (2.3)	2 (4.0)/1 (4.0)	0 (0)/0 (0)
AMP-DHS-CHL-SXT	1 (1.2)/1 (2.3)	1 (2.0)/1 (4.0)	0 (0)/0 (0)
AMP-DHS-SXT	16 (18.6)/13 (30.2)	8 (16.0)/8 (32.0)	8 (22.2)/5 (27.8)
AMP-DHS-CHL	1 (1.2)/1 (2.3)	1 (2.0)/1 (4.0)	0 (0)/0 (0)
AMP-DHS	10 (11.6)/6 (14.0)	0 (0)/0 (0)	10 (27.8)/6 (33.3)
DHS-SXT	1 (1.2)/1 (2.3)	0 (0)/0 (0)	1 (2.8)/1 (5.6)
AMP	1 (1.2)/1 (2.3)	1 (2.0)/1 (4.0)	0 (0)/0 (0)
DHS	10 (11.6)/7 (16.3)	7 (14.0)/5 (20.0)	3 (8.3)/2 (11.1)
OTC	1 (1.2)/1 (2.3)	1 (2.0)/1 (4.0)	0 (0)/0 (0)
NAL	1 (1.2)/1 (2.3)	1 (2.0)/1 (4.0)	0 (0)/0 (0)

Susceptible	21 (24.4)/13 (30.2)	12 (24.0)/7 (28.0)	9 (25.0)/6 (33.3)

^a^AMP, ampicillin; CFZ, cefazolin; CEF, ceftiofur; DHS, dihydrostreptomycin; GEN, gentamicin; KAN, kanamycin; OTC, oxytetracycline; CHL, chloramphenicol; NAL, nalidixic acid; ENR, enrofloxacin; SXT, trimethoprim-sulfamethoxazole.

**Figure 1:**
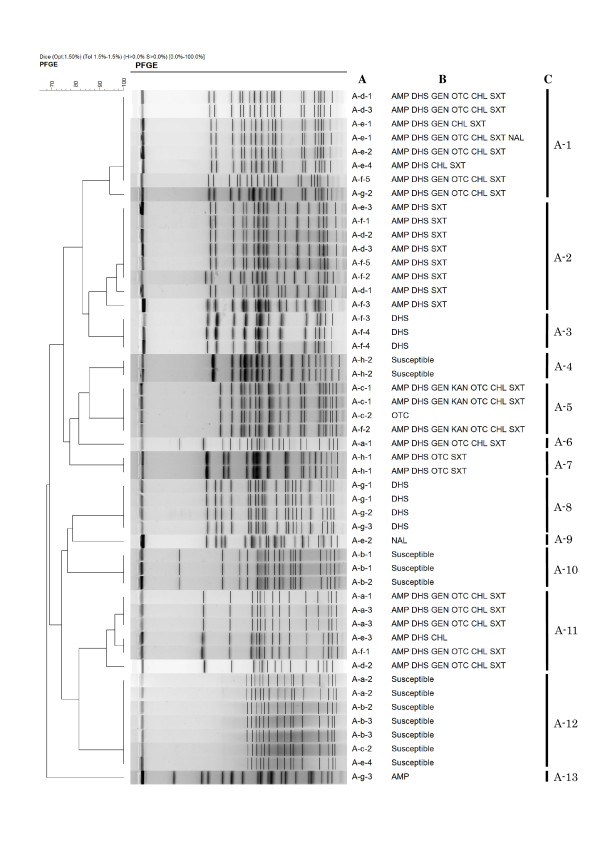
**Dendrogram of pulsed-field gel electrophoresis (PFGE) profiles from 50 *Escherichia coli *isolates from 25 pups originating from kennel A**. A: Isolate origin. A-a, A-b, A-c, A-d, A-e, A-f, A-g, and A-h litters consisted of three (A-a-1 to A-a-3), three (A-b-1 to A-b-3), two (A-c-1 to A-c-2), three (A-d-1 to A-d-3), four (A-e-1 to A-e-4), five (A-f-1 to A-f-5), three (A-g-1 to A-g-3), and two pups (A-h-1 to A-h-2), respectively. Two isolates were obtained per pup. B: Resistance pattern. AMP, ampicillin; DHS, dihydrostreptomycin; GEN, gentamicin; KAN, kanamycin; OTC, oxytetracycline; CHL, chloramphenicol; SXT, trimethoprim-sulfamethoxazole; NAL, nalidixic acid. C: PFGE profile.

**Figure 2:**
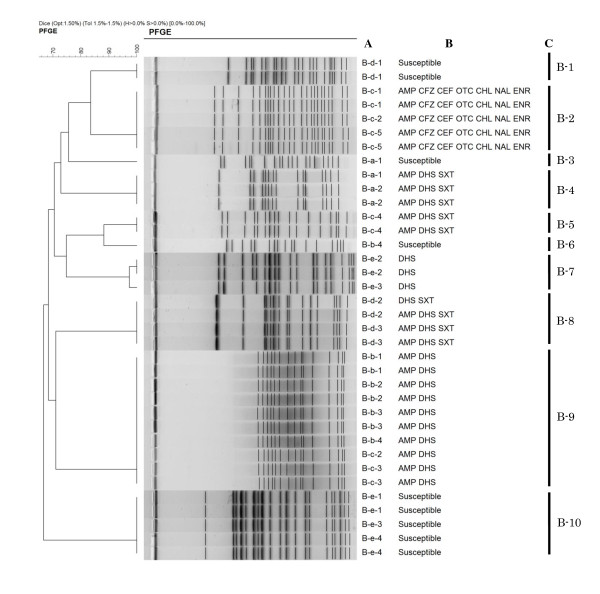
**Dendrogram of pulsed-field gel electrophoresis (PFGE) profiles from 36 *Escherichia coli *isolates from 18 pups originating from kennel B**. A: Isolate origin. B-a, B-b, B-c, B-d, and B-e litters consisted of two (B-a-1 to B-a-2), four (B-b-1 to B-b-4), five (B-c-1 to B-c-5), three (B-d-1 to B-d-3), and four pups (B-e-1 to B-e-4), respectively. Two isolates were obtained per pup. B: Resistance pattern. AMP, ampicillin; CFZ, cefazolin; CEF, ceftiofur; DHS, dihydrostreptomycin; GEN, gentamicin; OTC, oxytetracycline; CHL, chloramphenicol; SXT, trimethoprim-sulfamethoxazole; NAL, nalidixic acid; ENR, enrofloxacin. C: PFGE profile. Five isolates with the B-2 PFGE profile harboured the SHV-12 ESBL-encoding gene.

Additionally, the *Xba*I-digested PFGE revealed 13 and 10 distinct major profiles (≥ 90% Dice similarity) in 50 and 36 isolates from 25 and 18 pups from kennels A and B (Figures 1 and 2), respectively. These PFGE profiles correlated highly with resistance phenotypes, except for profiles A-1, A-5 and A-11. Of all 43 pups, 17 pups harboured two isolates differentiated by PFGE and/or resistance phenotypic profiles, indicating that diverse *E. coli *populations can colonise intestinal flora during infancy. Sixteen of 25 pups in kennel A (i.e. two, three, three, five, and three pups within litters A-a, A-b, A-d, A-f, and A-g, respectively) and 16 of 18 pups in kennel B (i.e. two, four, four, two, and four pups within from litters B-a to B-e, respectively) shared at least one *E. coli *clone, defined as an isolate with identical PFGE and resistance phenotypic profiles, with one or more pups of the same litters. There was no significant difference in the clone sharing rates within the same litters between the two kennels. In the two kennels, all pups of the same litters, and their respective dams, were raised together in one cage, implying that *E. coli *may be transmitted horizontally *via *faeces. Another possibility may be vertical transfer from mothers *via *their milk and vaginal flora [14]. These data suggest that pups from the same litter are likely to be exposed to common sources of *E. coli *resulting in clonal spread of organisms, including antimicrobial resistant isolates.

The following 24 pups in the two kennels shared at least one *E. coli *clone (i.e. the clones harbouring PFGE profiles A-1, 2, 5, 11, 12, and B-9) among the different litters; three, two, two, three, three, four, and one pups of from litters A-a to A-g in kennel A, and four and two pups of litters B-b and B-c, respectively. These litters were temporally separated (8-83 days) and originated from different mothers without direct contact, suggesting that *E. coli *clones may have originated from a persistent external source. One possibility, as suggested by other studies, is that the pups acquired *E. coli *from their human contacts [15,16]. Unlike clone sharing rates among the same litters, the rates among different litters were significantly different between kennels A and B [18/25 (72.0%) *vs. *6/18 (33.3%) pups, respectively, *P *< 0.05]. This finding suggests that clone sharing rates among different litters can vary between kennels. Further study is needed to clarify the potential transmission route(s) between kennel pups. Overall, our data indicates that clonal spread of *E. coli *plays an important role in acquisition of resistant isolates by kennel pups.

The prevalence of ESBL-producing isolates in companion animals and their potential impact on human health is a major issue [17]. In the present study, the SHV-12 ESBL-encoding gene was detected in five isolates (5.8%), exhibiting identical PFGE and resistance phenotypic profiles, from three pups within a litter (Figure 2). The reason for the occurrence of these resistant isolates was not apparent. To the best of our knowledge, this is the first time that SHV-12 β-lactamase has been detected in *E. coli *of canine origin in Japan, although it has been previously reported in other countries [18,19]. The present findings suggest that attention needs to be paid to dogs as a potential reservoir of ESBL-producing *E. coli *isolates in Japan.

In conclusion, our data show that pups in kennels can harbour multidrug-resistant *E. coli *isolates, including ESBL-producing isolates. The present results also indicate that resistant and susceptible *E. coli *isolates can clonally spread not only within the same litter but also among different litters thus affecting the prevalence of resistant organisms in a kennel. Further studies are needed to fully understand the epidemiological spread of antimicrobial resistant bacteria among pups in kennels.

## Competing interests

The authors declare that they have no competing interests.

## Authors' contributions

KH and EM carried out antimicrobial susceptibility testing, PCR, sequencing, and PFGE. KH analysed the data. KH, YK and TT were involved in the study design and preparation of the manuscript. KH drafted the manuscript. All authors read and approved the final manuscript.
